# SRSF1 promotes the inclusion of exon 3 of *SRA1* and the invasion of hepatocellular carcinoma cells by interacting with exon 3 of SRA1pre-mRNA

**DOI:** 10.1038/s41420-021-00498-w

**Published:** 2021-05-19

**Authors:** Sijia Lei, Bin Zhang, Luyuan Huang, Ziyou Zheng, Shaohan Xie, Lianghua Shen, Mason Breitzig, Alexander Czachor, Hongtao Liu, Huiru Luo, Yanxia Chen, Kangshou Liu, Hanxiao Sun, Qing Zheng, Qiang Li, Feng Wang

**Affiliations:** 1 Institute of Genomic Medicine, College of Pharmacy, Jinan University, Guangzhou, China; 2 International Cooperative Laboratory of Traditional Chinese Medicine Modernization and Innovative Drug Development of Chinese Ministry of Education (MOE), College of Pharmacy, Jinan University, Guangzhou, China; 3 University of Chinese Academy of Science, Beijing, China; 4 Department of Internal Medicine, Morsani College of Medicine, University of South Florida, Tampa, FL USA; 5 Brown School of Social Work, Washington University in St. Louis, St. Louis, MO USA; 6 College of Life Sciences, Zhengzhou University, Zhengzhou, Henan China; 7 Department of General Surgery, The First Affiliated Hospital, Jinan University, Guangzhou, China

**Keywords:** Diagnostic markers, Molecular biology

## Abstract

Steroid receptor RNA activator 1 (*SRA1*) has been described as a novel transcriptional co-activator that affects the migration of cancer cells. Through RT-PCR, we identified that skipping exon 3 of SRA1 produces two isoforms, including the truncated short isoform, SRA1-S, and the long isoform, SRA1-L. However, the effect of these two isomers on the migration of HCC cells, as well as the specific mechanism of exon 3 skipping remain unclear. In this study, we found up regulated expression of SRSF1 and SRA1-L in highly metastatic HCCLM3, as well as in HCCs with SRSF1 demonstrating the strongest correlation with SRA1-L. In contrast, we observed a constitutively low expression of SRA1-S and SRSF1 in lowly metastatic HepG2 cells. Overexpression of SRSF1 or SRA1-L promoted migration and invasion by increasing the expression of CD44, while SRA1-S reversed the effect of SRSF1 and SRA1-L in vitro. In addition, lung metastasis in mice revealed that, knockdown of *SRSF1* or *SRA1-L* inhibited the migration of HCC cells, while *SRA1-L* overexpression abolished the effect of *SRSF1* knockout and instead promoted HCC cells migration in vivo. More importantly, RNA immunoprecipitation and Cross-link immunoprecipitation analyses showed that SRSF1 interacts with exon 3 of *SRA1* to up regulate the expression of SRA1-L in HCC cells. RNA pull-down results indicated that SRSF1 could also bind to exon 3 of SRA1 in vitro. Finally, minigene -MS2 mutation experiments showed that mutation of the SRA1 exon 3 binding site for SRSF1 prevented the binding of SRA1 pre-mRNA. In summary, our results provide experimental evidence that SRA1 exon 3 inclusion is up regulated by SRSF1 to promote tumor invasion and metastasis in hepatocellular carcinoma.

## Introduction

Liver cancer is a common cancer in the world. Many deaths of cancer patients are caused by liver cancer^1^. In 90% of solid cancer cases, metastasis is the main reason for mortality^2^. It can be seen that inhibiting tumor metastasis is an effective way to improve the survival rate of patients. More specifically, studies have shown that alternative splicing (AS) plays an important role in cancer metastasis^3–6^. Although many abnormal alternative splicing events of mRNA have been reported, few articles have reported on alternative splicing events that simultaneously produce-coding proteins isoforms and non-coding isoforms^7^. Therefore, further study of alternative splicing will improve our understanding of liver cancer metastasis and promote the development of more effective targeted therapies.

*SRA1* was first described as a novel transcriptional co-activator in 1999 (Accession number: AF092038) and originally characterized as a non-coding RNA^8^ responsible for transcriptional co-activation of steroid hormone receptors^9^. Further investigations identified additional *SRA1* ncRNA isoforms produced by alternative splicing or multiple transcription start sites^10^. Currently, there are three main isoforms of *SRA1* ncRNA: (1) retention of intron 1, (2) alternative 3′ splicing site of exon 2, and (3) deletion of exon 3^10–12^. Interestingly, in HCC cells, we only detected the isoform with the deletion of exon 3 in LO2, HepG2, and HCCLM3 cells.

Previous studies suggested that SRA-mediated co-activation is executed by distinct RNA motifs. These studies identified five substructures, which were important for *SRA1* co-activation^9^. The deletion of exon 3 would disrupt the formation of two RNA motifs: STR-9 and STR-10. *SRA1-S*, with a deletion of exon 3 of *SRA1-L*, mediated the ability of co-activation probably unlike *SRA1-L*. Therefore, whether *SRA1-L* and *SRA1-S* have different functions due to the deletion of exon 3 needs to be further explored. Also, *SRA1* stimulates proliferation and apoptosis in vivo^13^. At present, it is not clear whether there are functional differences between *SRA1-L* and *SRA1-S* in the proliferation and metastasis of hepatocellular carcinoma cells.

The SR splicing factor family consists of 12 members. Each member contains one or two RNA recognition motifs (RRM) and an RS domain. SR protein can participate in splicing, and can also participate in RNA transcription, export, translation and degradation^14^. SRSF1 is a member of SR splicing factor family. The expression of SRSF1 is related to the occurrence, development and treatment response of tumor, and SRSF1 is highly expressed in tumor cells^15^. SRSF1 can affect tumorigenesis by regulating the alternative splicing of target genes. For examble, For example, SRSF1 regulates PTPMT1 alternative splicing to affect the development of lung cancer^16^. SRSF1 can also regulate BIN1 alternative splicing to affect tumorigenesis^17^. In this study, we found that SRSF1 can regulate the alternative splicing of SRA1 in hepatocellular carcinoma cells, but the specific mechanism is not clear.

Oxisome proliferator-activated receptor (peroxisome proliferators-activated receptor, PPAR) is a kind of nuclear transcription factors activated by ligands, which belongs to the nuclear receptor superfamily. Many articles have reported that PPAR-γ is related to the occurrence and development of tumors^18–20^. It is reported that *SRA1* can combine with PPAR-γ^21^. At present, it has not been found whether there is a difference in the binding ability of *SRA1-L* and *SRA1-S* with PPAR-γ.

Here, we used cell migration experiment and nude mouse metastasis model to determine the role of SRSF1 and SRA1 in HCC cells metastasis. At the same time, we verified the potential binding sites of SRSF1 and *SRA1* pre-mRNA through RNA pull-down and other experiments. In addition, through immunoprecipitation, we found that SRA1 isoforms (*SRA1-L*, *SRA1-S*) have different binding capabilities with PPAR-γ. Finally, our results confirmed that SRSF1 is a protein associated with metastasis, and SRA1 is the splicing target of SRSF1, which regulates the incorporation of exon3 to promote lung cancer cell metastasis. Understanding the mechanism underlying the alternative splicing of *SRA1* can provide novel insight into the identification of new therapeutic targets.

## Results

### SRA1-L expression was increased in higher-metastatic HCC cells

**Fig. 1:**
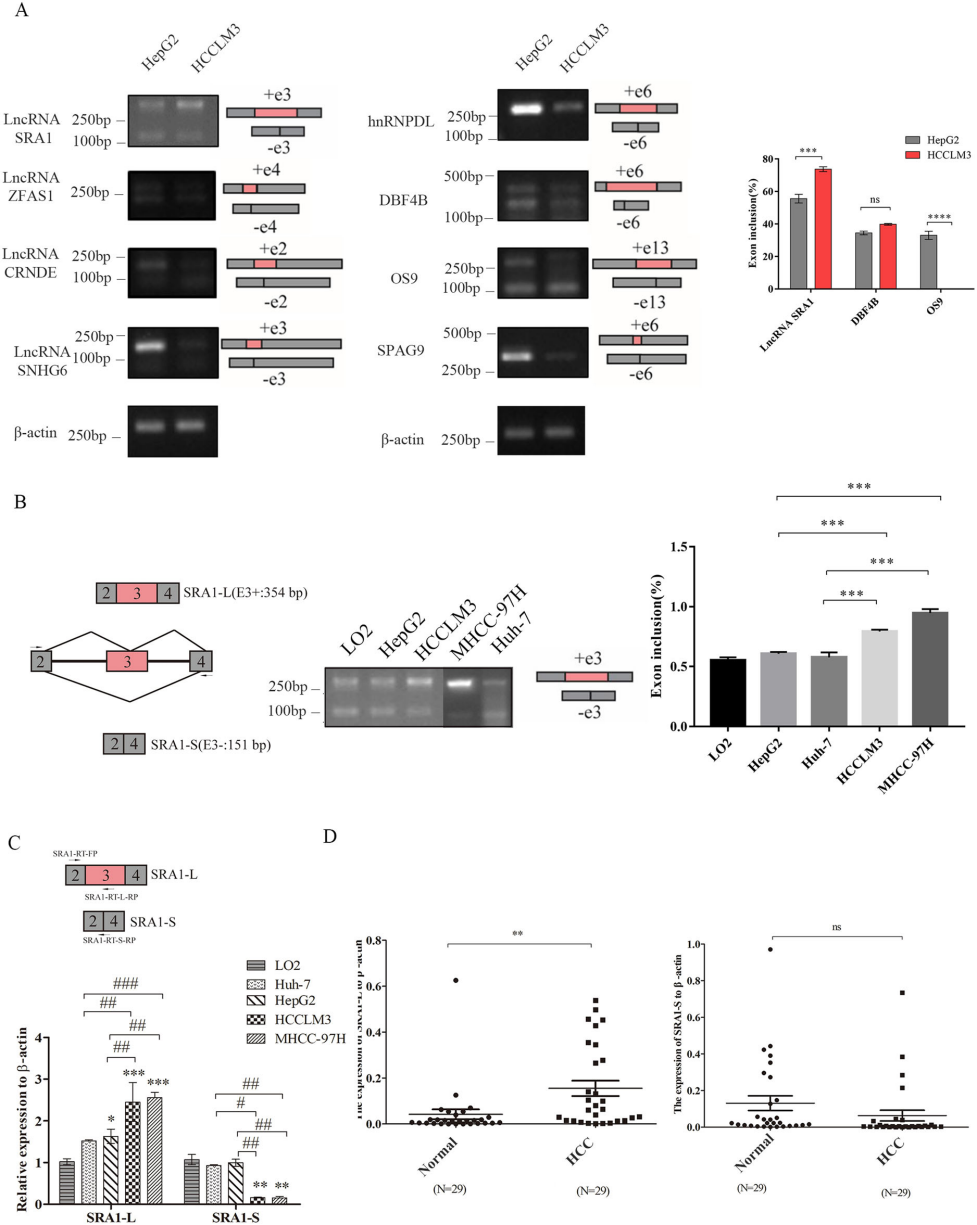
Differential expression of *SRA1* isoforms in Hepatocellular carcinoma cells. **A** Alternative splicing of the different genes in HepG2 and HCCLM3 by RT-PCR. **B** The expression of *SRA1-L* was examined in LO2, Huh-7, HepG2 and HCCLM3, MHCC-97H by RT-PCR. **C** The expression of *SRA1-L* was examined in LO2, Huh-7, HepG2 and HCCLM3, MHCC-97H by RT-qPCR. **D** RT-qPCR was used to detect the expression of *SRA1-L* and *SRA1-S* in HCCs. Data from the experiments with one experimental group were presented as means ± SD (all experiments were performed in triplicate or more). Data are presented as mean ± S.D. (*N* = 3). The “*, **, ***” indicate “*P* < 0.05, 0.01, 0.001” versus the control group, respectively.

Firstly, we selected several genes related to tumor metastasis through the NCBI database and literature, including *SRA1*, DBF4 zinc finger B (*DBF4B*), ZNFX1 antisense RNA 1 (*ZFAS1*), colorectal neoplasia differentially expressed (*CRNDE*), endoplasmic reticulum lectin (*OS9*), and others. Then, we detected the alternative splicing of these genes in HepG2 and HCCLM3 cells by RT-PCR. The results showed that compared with HepG2 cells, in HCCLM3 cells, the exon inclusion rate of *DBF4B* did not change significantly. Only the long isomer of *hnRNPDL*, *SNHG6* and the short isomer of *SPAG9* were detected in HCC cells. More importantly, the SRA1 exon 3 inclusion rate of HCCLM3 cells was significantly higher than that of HepG2 cells (Fig. 1A). The results of RT-PCR and qPCR also showed that in high-metastatic HCC cells, including HCCLM3 and MHCC-97H, the proportion of *SRA1-L* isoforms was higher than that of low-metastasis HepG2 HCC cells and normal liver cells (LO2) (Fig. 1B, C). From the detection of clinical samples of HCCs, we also found that the expression of *SRA1-L* in HCCs was higher than that in adjacent tissues (Fig. 1D).

### In contrast to *SRA1*-S, *SRA1*-L promotes the migration of HCC cells

**Fig. 2:**
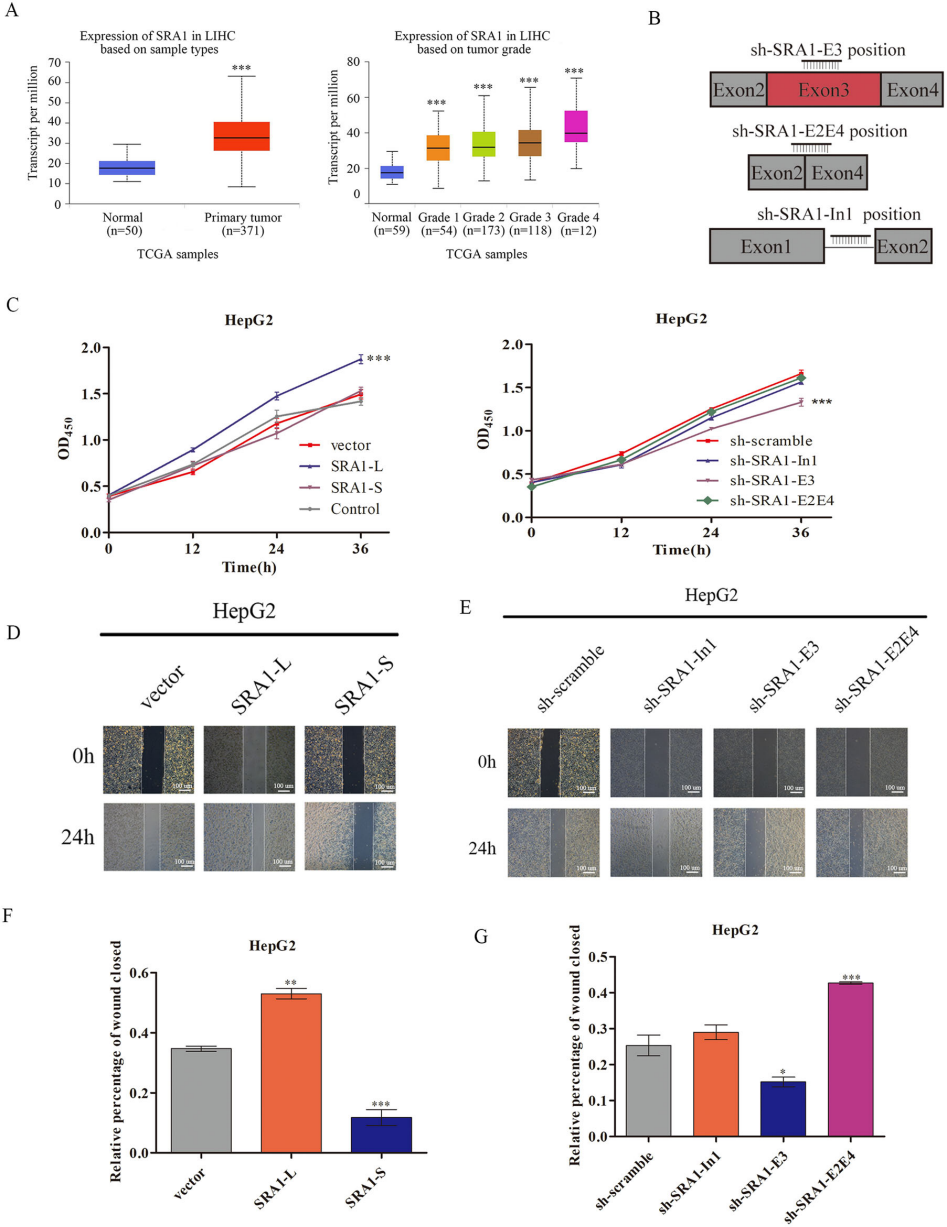
The isoforms of SRA1 have different effects. **A** SRA1 expression levels in human HCC tissues from TCGA samples. **B** Design of shRNA applied to selectively target the In1, exon 3 and exon 2/exon 3 splice junctions. **C** CCK8 assays were carried out to examine the effect on cell proliferation of *SRA1-L/S* in HepG2. **D**, **F** Wound healing experiments were carried out to estimate the effects of overexpressed *SRA1-L/S* on the migration in HepG2. **E**, **G** Wound healing experiments were carried out to estimate the effects of knocked-down SRA1-E3, SRA1-E2E4, and SRA1-In1 on the migration in HepG2. Data are presented as mean ± S.D. (*N* = 3). The “*, **, ***” indicate “P < 0.05, 0.01, 0.001” versus the control group, respectively. “^#^, ^##^, ^###^” represents “*P* < 0.05, 0.01, 0.001”.

*SRA1* has been reported to regulate cell invasion and migration^22,23^. We posited that the two *SRA1* isoforms might have different effects. To test this, we first analyzed *SRA1* expression in 371 liver hepatocellular carcinoma (LIHC) tissues and 50 normal tissues from UALCAN database^24^. Expression of SRA1 was found to significantly increase in liver cancer (Fig. 2A). Subsequently, we overexpressed *SRA1-L* and *SRA1-S*, and designed an independent shRNA targeted against exon 3 for isoform-specific knockdown of *SRA1-L*; shRNA targeted against exon 2/exon 4 (E2E4) splice junction for isoform-specific knockdown of *SRA1-S* and an shRNA to selectively target the Intron 1 in HepG2 cells (Fig. 2B). The overexpression and knockdown efficiency of SRA1 isomers have been verified (Fig. S5A, B). In cell proliferation, the results showed that knockdown of *SRA1-L* could significantly inhibit the proliferation of HepG2 cells (Fig. 2C). Increasing the expression of *SRA1-L* can significantly promote the proliferation of HepG2 cells. Also, overexpression or the depletion of *SRA1-S* has no significant effects on cell proliferation of HepG2 cells (Fig. 2C). The results of wound healing showed overexpressed *SRA1-L* promoted the migratory abilities of HepG2 cells, while overexpressed *SRA1-S* inhibited the migratory abilities of HepG2 cell (Fig. 2D–F). Consistent with the above results, knockdown *SRA1-L* inhibits the migratory abilities of HepG2 cell, meanwhile, knockdown *SRA1-S* promotes the migratory abilities of HepG2. (Fig. 2E, G). In cell invasion, knockdown of *SRA1-L* significantly decreased the invasive ability of hepatoma cells while knockdown of SRA1-In1 had no significant impact. Meanwhile, knockdown of *SRA1-S* increased the invasive ability of hepatoma cells (Fig. 5E, G). Over-expressing *SRA1-L* increased the invasive ability of hepatoma cells, but over-expressing *SRA1-S* decreased the invasive ability of hepatoma cells (Fig. 5F, H).

### SRA1-L promotes the migration of HCC cells by increasing the level of CD44 transcription

**Fig. 3:**
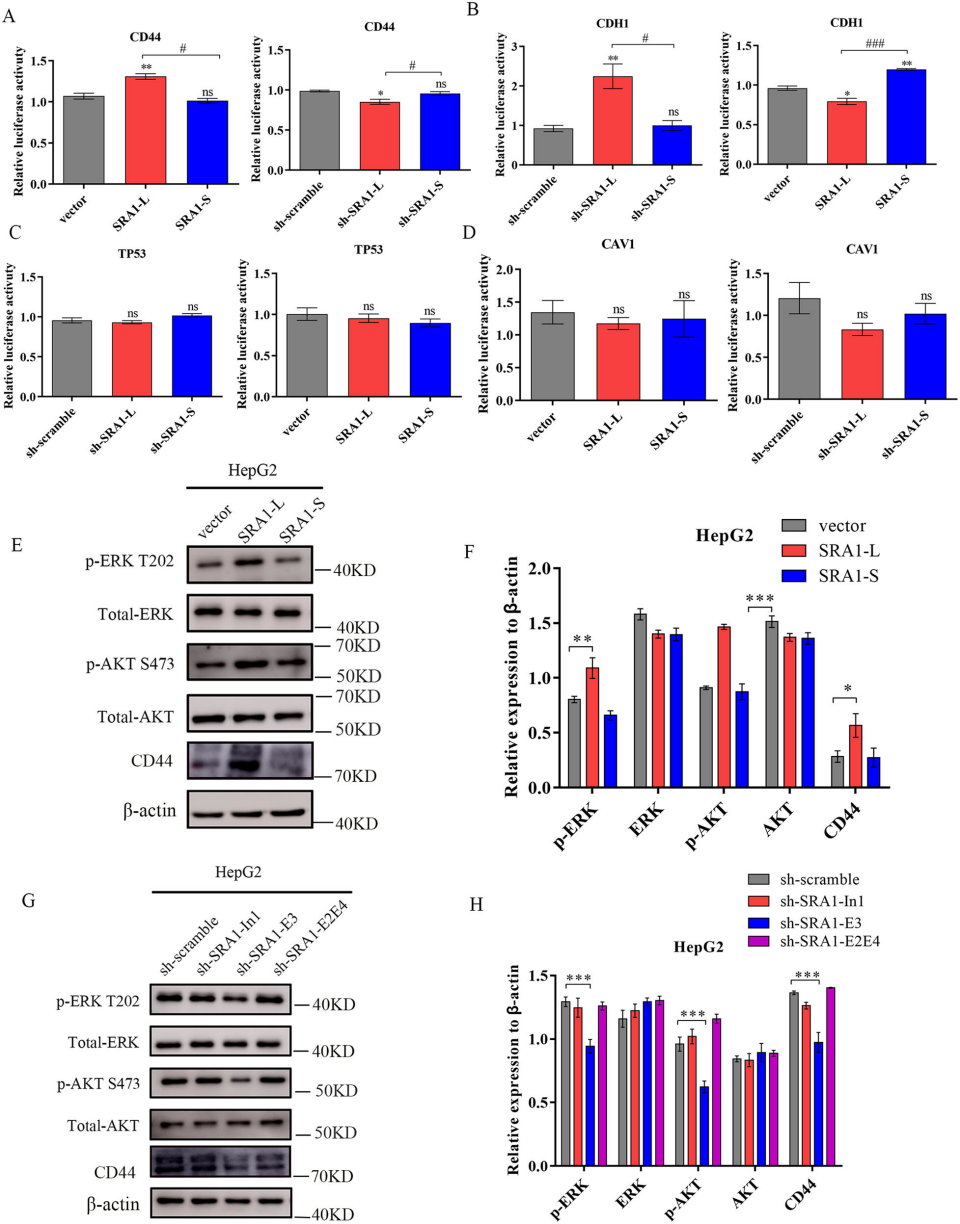
SRA1 isoforms have different regulatory effects on gene transcription. **A** The effect of SRA1 on CD44 Transcription. **B** The effect of SRA1 on CDH1 Transcription. **C** The effect of SRA1 on TP53 Transcription. **D** The effect of SRA1 on CAV1 Transcription. **E**, **F** The effect of overexpressed *SRA1-L/S* on downstream CD44, AKT, and ERK signaling pathway proteins was detected in HepG2 cells by western-blot. **G**, **H** The effect of knocked down SRA1-E3, SRA1-E2E4, and SRA1-In1 on downstream CD44, AKT, and ERK signaling pathway proteins was detected in HepG2 cells by western-blot. Data are presented as mean ± S.D. (*N* = 3). The “*, **, ***” indicate “*P* < 0.05, 0.01, 0.001” versus the control group, respectively. “^#^, ^##^, ^###^” represents “*P* < 0.05, 0.01, 0.001”.

SRA1 was a novel transcriptional co-activator, which can regulate the transcription of target genes. Our result of Luciferase Reporter Assays shows that *SRA1-L* can promote the transcription of *CD44*, but *SRA1-S* has no significant effect on the transcription of *CD44* (Fig. 3A). According to the literature, CD44 can promote tumor metastasis through the regulation of ERK and AKT pathway^25,26^, and our results indicate that over-expression of *SRA1-L* promotes the expression of p-AKT, CD44 and p-ERK, knockdown *SRA1-L* decreased the expression of p-AKT, CD44 and p-ERK. But *SRA1-S* does not affect on the expression of p-AKT, CD44 and p-ERK (Fig. 3E–H).

In addition, Luciferase Reporter Assays shows that *SRA1-L* can inhibit the transcription of *CDH1*, knockdown *SRA1-L* decreased the transcription of *CDH1* (Fig. 3B).

### SRSF1 is the major regulator for SRA1 exon 3 splicing

**Fig. 4:**
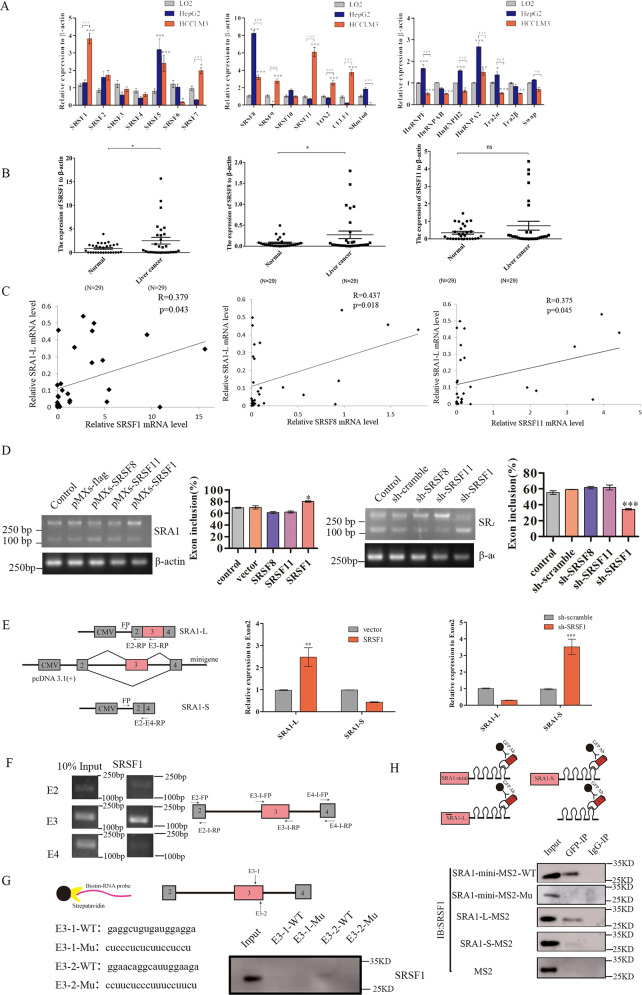
SRSF1 regulates alternative splicing of SRA1 exon 3 by binding to “GGAACAGGCATTGGAAGA” sequence in exon 3 to promote exon inclusion. **A** RT-qPCR was used to detect the expression levels of different splicing factors in LO2, HepG2, and HCCLM3 cells. **B** Detect the mRNA expression of SRSF1, SRSF8, SRSF11 and SRA1-L in HCCs. **C** Correlation analysis of the expression levels of SRSF1, SRSF8, SRSF11 and SRA1-L. Use SPSS analysis tools for correlation analysis of gene expression. **D** Alternative splicing of exon 3 of SRA1 was examined in HCCLM3 with over-expression or knockdown of SRSF1, SRSF8, and SRSF11 by RT-PCR. **E** Graphical and specific primer design sites for pcDNA3.1-SRA1-minigene. The primers for the internal reference of exogenous SRA1-minigene were CMV-FP (FP), exon 2-RP (E2-RP), exon 3-RP (E3-RP) and exon 2/exon 3 splice junction-RP (E2-E4-RP), and the primers for RT-qPCR detection of exogenous SRA1 isoforms in HCCLM3. **F** The primers used to detect the binding segment of SRSF1 and its positions in SRA1 pre-mRNA in the CLIP experiment. The CLIP assays were applied to detect the binding between SRSF1 and SRA1 pre-mRNA. **G** The RNA fragment sequences utilized in the RNA pulldown assay and its positions in the SRA1 pre-mRNA. RNA pulldown assays were used to detect the binding between SRSF1 and the biotinylated SRA1 RNA fragments. **H** Graphical representation of the MS2-GFP-IP system and its mode of operation. The effect of SRSF1 binding to SRA1 was investigated by the MS2-GFP-IP system. Data are presented as mean ± S.D. (*N* = 3). The “*, **, ***” indicate “*P* < 0.05, 0.01, 0.001” versus the control group, respectively.

SR proteins and hnRNP proteins are well-known splicing factors that have major roles in the regulation of AS. To find the potential splicing factors regulating the alternative splicing of exon 3 of SRA1, firstly, using public datasets from UALCAN^24^, we examined the connection between the expression of splicing factors and the major cancer stages of liver cancer. Compared with the normal group, the expression of SRSF1, SRSF11 was found to significantly increase in the primary tumor of liver cancer (Fig. S1A). Then, we examined the expression of the SRSF family, the hnRNP family, and other splicing factors in LO2, HepG2, and HCCLM3 cells by RT-qPCR. The results showed that the expression of SRSF1, and SRSF11 in HCCLM3 was significantly higher than that of HepG2 and LO2 (Fig. 4A). Also, in 29 clinical samples of HCCs, we detected the mRNA expression of SRSF1, SRSF8, and SRSF11. The results showed that the expression of SRSF1 in HCCs was higher than that in normal tissue (Fig. 4B). Then, in order to investigate whether SRSF1, and SRSF11 have an effect on the alternative splicing of SRA1. We analyzed the correlation between the expression levels of SRSF1, and SRSF11 with *SRA1*-L in 29 pairs of HCCs. The results show that the expression levels of SRSF1, and SRSF11 are positively correlated with SRA1-L. The correlation coefficient of SRSF1 is 0.379 (*p* < 0.05), the correlation coefficient of SRSF8 is 0.437 (*p* < 0.02), and the correlation coefficient of SRSF11 is 0.375 (*p* < 0.05) (Fig. 4C). Therefore, we preliminarily inferred that SRSF1, SRSF11 may be the upstream regulator of SRA1. Also, western-blot results indicate that the expression of SRSF1 in HCCLM3 cells was significantly higher than that in normal LO2 cells and low-transformed hepatoma cells, HepG2 and Huh-7 (Fig. S1B). Then, we overexpressed and knocked down SRSF1, and SRSF11 in HCCLM3 cells. We employed RT-qPCR to detect the overexpression and knockdown efficiency of SRSF1, and SRSF11 (Fig. S2A, B and Fig. S3A, B). We found that knockdown of SRSF1 significantly increased the expression of *SRA1-S* while the over-expression SRSF1 significantly increased the expression of *SRA1-L* in HCCLM3 cells. SRSF8 and SRSF11 had no significant effect on the alternative splicing of SRA1 in HCCLM3 cells (Fig. 4D).

Also, we measured the expression of the long isoforms of SRA1 in cells stably transduced with SRSF1 and deletion mutants RRM1, RRM2 and RS by RT-PCR. We found that the overexpression of SRSF1 can promote the inclusion of exon 3 of SRA1 compared with the empty vector control. ΔRS and ΔRRM2 mutants promoted a similar increase in the inclusion of exon 3, while ΔRRM1 mutants didn’t increase the inclusion of exon 3 of *SRA1* (Fig. S4A). These results suggest that SRSF1 can regulate the alternative splicing of SRA1 and promote the production of *SRA1-L*.

### SRSF1 enhances splicing of SRA1 exon 3 through its interaction with “GGAACAGGCAUUGGAAGA” sequence in exon 3

To further illustrate that SRSF1 is involved in the alternative splicing of SRA1 pre-mRNA, we constructed an SRA1-minigene vector. We transfected SRA1-minigene into HCCLM3 cells overexpressing SRSF1 and knocking down SRSF1. Then we detected the expression of exogenous SRA1-L and SRA1-S by RT-PCR. The results of RT-qPCR (Fig. 4E) showed that *SRA1-S* in minigene was significantly increased in HCC cells where SRSF1 was knocked down compared with non-treated cells. Consistent with expectations, we observed the opposite result in HCC cells by overexpressing SRSF1. Overexpression of SRSF1 promoted the generation of *SRA1-L*.

**Fig. 5:**
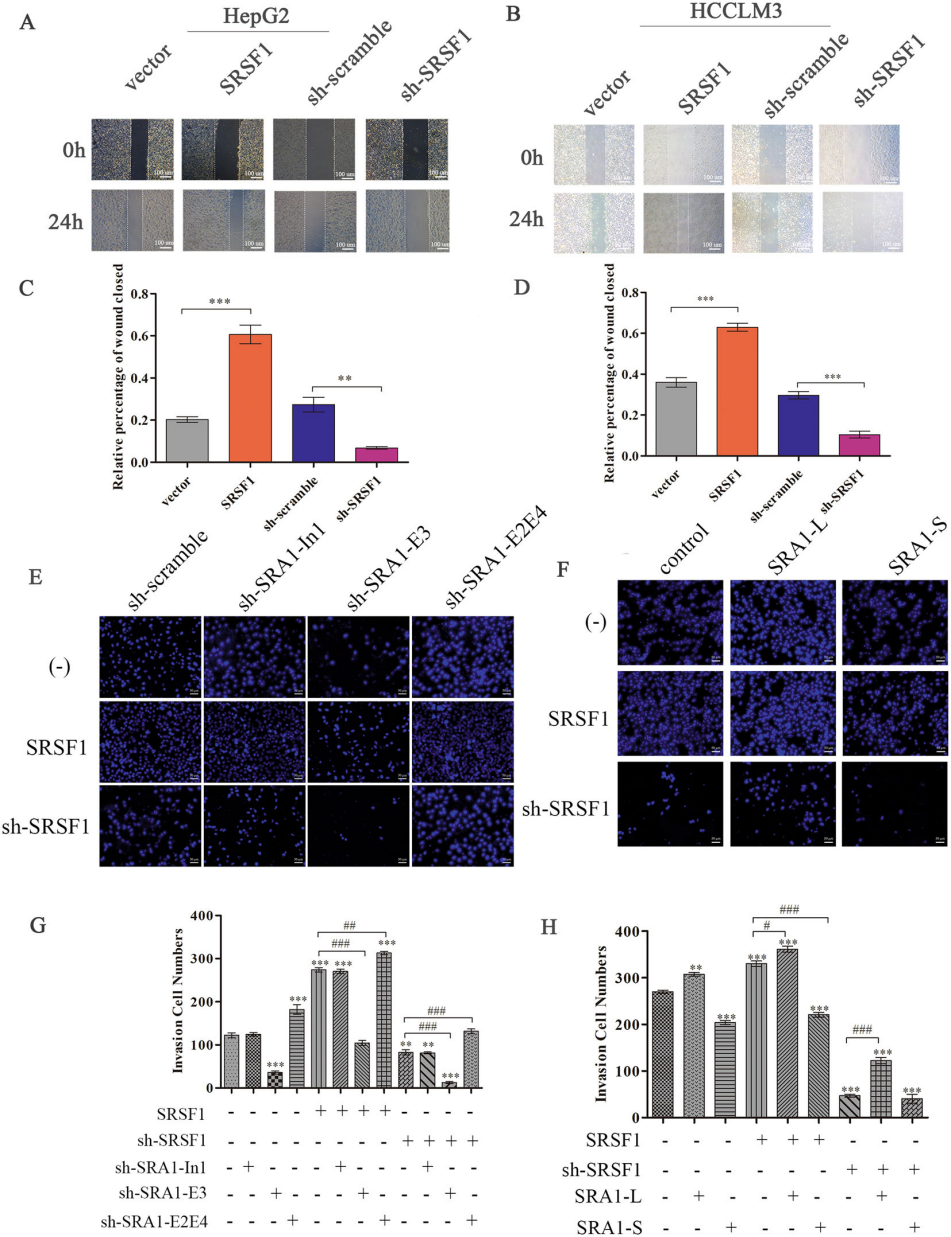
SRSF1 promotes the migration of HCC cells partially via regulating SRA1. **A**, **C** Wound healing experiments were carried out to estimate the effects of SRSF1 on the migration in HepG2 and HCCLM3. **B**, **D** Wound healing experiments were carried out to estimate the effects of SRSF1 on the migration in HCCLM3. The magnification of Wound healing experiments is 100× and the scale length is 100 μm. **E**–**H** The effect of SRSF1, *SRA1-L,* and *SRA1-S* expression levels on the invasive ability of HepG2 cells was examined by trans-well assays. The magnification of trans-well assays is 200× and the scale length is 50 μm. Data are presented as mean ± S.D. (*N* = 3). The “*, **, ***” indicate “*P* < 0.05, 0.01, 0.001” versus the control group, respectively.

SRSF1 is a typical splicing factor that preferentially binds to exonic sequences to promote exon inclusion^27^. Then we found that GAAGA^28,29^ and GGAGGA^27,30^ sequence was present in the exon3 of SRA1. So, we designed primers in exon 2, exon 3, and exon 4, and performed CLIP to detect whether SRSF1 binds to exon 3. The result of RT-PCR indicated that the binding of SRSF1 to exon 3 was stronger than others (Fig. 4F). At the same time, we verified whether SRSF1 and *SRA1* were bound by MS2-RIP experiments. The result of MS2-RIP show that SRSF1 and SRA1 pre-mRNA are bound before splicing (Fig. 4H). Through the analysis of the SRA1 base sequence, we found two potential binding sequences for SRSF1: (GAGGCTGTGATGGAGGA, E3-1) and (GGAACAGGCATTGGAAGA, E3-2) in exon 3 (Fig. 5G). To determine the interactions between the two potential binding sites in exon 3 and SRSF1, RNA-pulldown assays were performed. We synthesized biotinylated SRA1 RNA oligomers and the mutations of potential binding sites. The GAGGCUGUGAUGGAGGA (E3-1-WT) sequence in exon 3 was mutated to CUCCCUCUCUUCCUCCU (E3-1-Mu). The GGAACAGGCAUUGGAAGA (E3-2-WT) sequence in exon 3 was mutated to CCUUCUCCCUUUCCUUCU (E3-2-Mu). Data showed that SRSF1 could combine with E3-2-WT, but not E3-1-WT, E3-1-Mu, or E3-2-Mu (Fig. 4G). At the same time, we mutated “GGAACAGGCAttGGAAGA” in SRA1-mini-MS2-WT to “ggaCcCggcCttggaaga” (SRA1-mini-MS2-Mu). When the binding site of SRSF1 in the minigene was artificially mutated, SRSF1 could not bind to the pre-mRNA of SRA1(Fig. 4H). The above results indicated that SRSF1 promotes the inclusion of exon 3 by binding to the “GGAACAGGCATTGGAAGA” sequence in exon 3, thus regulating the alternative splicing of exon 3 of SRA1.

### SRSF1 promoting the migration of HCC cells partially depends on the *SRA1-L*

The above research results indicated that SRSF1 can regulate the alternative splicing of *SRA1* and promote the expression of *SRA1-L*. Also, SRA1-L and SRA1-S can affect the migration of HCC cells. Our results of wound healing showed that overexpressed SRSF1 can increase the migratory abilities of HepG2 and HCCLM3 cells. Meanwhile, the depletion of SRSF1 significantly inhibited this ability (Fig. 5A, B). Also, trans-well experiments showed that overexpression of SRSF1 enhanced the ability of cell migration, and the depletion of SRSF1 significantly inhibited the ability of cell migration (Fig. 5E, F). And overexpression of SRSF1 and knockout of SRA1-L reduced the invasion of hepatoma cells, compared with HepG2 cells with overexpression of SRSF1 alone (Fig. 5E). Similarly, knockdown SRSF1 and overexpress SRA1-L are more aggressive than knockdown SRSF1 alone (Fig. 5F).

**Fig. 6:**
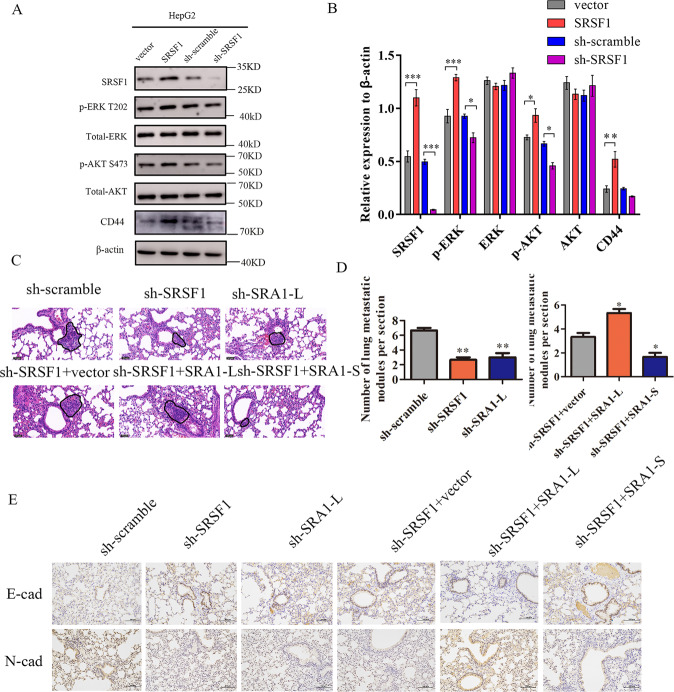
In vivo, SRSF1 promotes the metastasis of HCC cells. **A**, **B** The effect of SRA1 on downstream CD44, AKT, and ERK signaling pathway proteins was detected in HepG2 cells by western blot. **C**, **D** HE staining was used to analyze the number of pulmonary metastases in different groups of nude mice. The magnification of HE is 200× and the scale length is 50 μm. **E** The expression level of N-cad and E-cad in tumor tissues was analyzed by immunohistochemistry. The magnification of immunohistochemistry is 100× and the scale length is 100 μm. Data are presented as mean ± S.D. (*N* = 3). The “*, **, ***” indicate “*P* < 0.05, 0.01, 0.001” versus the control group, respectively.

Western-blot results indicate that over-expression of SRSF1 can promote the expression of p-AKT, CD44, and p-ERK, and the depletion of SRSF1 significantly inhibits the expression of p-AKT, and p-ERK (Fig. 6A, B). In addition, we assessed the effects of SRSF1 overexpression or depletion on cell proliferation. The results of CCK8 showed that knocking down SRSF1 inhibited the proliferation of HepG2 and HCCLM3 cells. Overexpression of SRSF1 promoted the proliferation of HepG2 and HCCLM3 cells (Fig. S4B).

In vivo metastasis assays, HE staining showed that the lung metastatic nodules of nude mice were significantly reduced after knocking out SRSF1 or SRA1-L (Fig. 6C, D). Interestingly, the overexpression of SRA1-L reversed the inhibitory effect of down-regulated SRSF1 on lung metastasis of hepatocellular carcinoma cells. In addition, compared with the control group, the inhibitory effect of sh-SRSF1 + SRA1-S on lung metastasis of hepatocellular carcinoma cells was stronger in the experimental group (Fig. 6C, D). Immunohistochemistry showed that knockdown of *SRA1-L* or SRSF1 significantly reduced the expression of N-cad, while knockdown of *SRA1-L* or SRSF1 significantly increased the expression of E-cad (Fig. 6E). Excitingly, overexpression of *SRA1-L* significantly reversed the effect of knockdown of SRSF1 on the expression of E-cad and N-cad. Besides, compared with the control group, the group of shSRSF1 and overexpression of *SRA1-S* more robustly inhibited the expression of N-cad and promoted E-cad (Fig. 6E). Therefore. SRSF1 may affect the metastasis of HCC by regulating the splicing of SRA1.

## Discussion

**Fig. 7:**
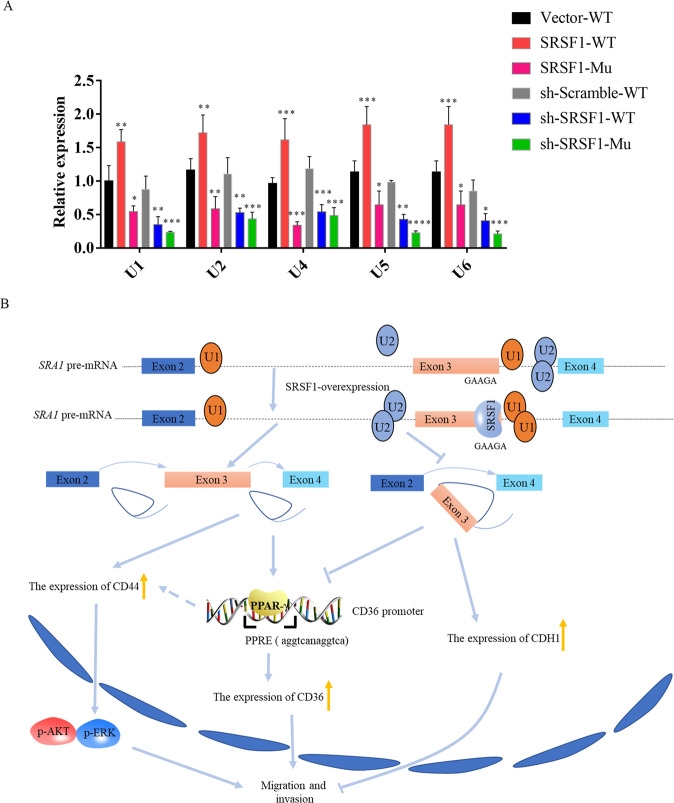
SRSF1 regulates the alternative splicing of SRA1 and promotes the metastasis of liver cancer. **A** The effect of SRSF1 on the ability of snRNAs to bind to SRA1 pre-mRNA was studied by the MS2-GFP-RIP system. **B** The effect of the SRSF1-SRA1 signal axis on the migration and invasion of the HCC cells.

Abnormal alternative splicing is often detected in the occurrence and development of hepatocellular carcinoma^31^. For examble, Zhang et al found that hnRNPU regulates the alternative splicing of DIS3L2 to promote the development of liver cancer^32^. Friedman et al found that KLF6 regulates the alternative splicing of Ras to promote the proliferation of hepatocellular carcinoma cells^33^. In this study, We found that SRSF1 regulates the alternative splicing of SRA1 to promote the migration of hepatocellular carcinoma cells. Both SRSF1 and *SRA1-L* enhance cell growth, invasion, and migration in HCC cells (Fig. 7B).

The recognition of RNA binding sites by SRSF1 varies, with binding sequences including: RGAAGAAC, AGGACAGAGC, AGGACRRAGC, AACAGGACAA, AA(AGGACAA)_2_AA, SRSASGA (S = G/C, R = A/G)^30,34,35^. In this paper, we found that SRSF1 regulates the splicing of SRA1 by binding to “GGAACAGGCAUUGGAAGA” on exon 3 of SRA1. Combined with the results of this article, we compared the sequence characteristics of SRSF1 preference binding, and found that the AG content in the sequence is higher, and the sequence AG seems to be interlaced. So, study of the pre-mRNA recognition site of SRSF1 could provide a molecular mechanism for examining the SRSF1- mediated splicing of target genes.

There are many hypotheses about the molecular mechanism of SRSF1-mediated splicing regulation. It has been reported that SR protein binds to exon ESE, directs U2AF protein to 3 ‘splice site and U1snRNP to 5′ splice site^36–38^. Also, SR protein can promote splice assembly^39^. MS2-IP experiment shows that overexpression of SRSF1 increases the binding of U1-U6 to pre-mRNA (Fig. 7A). SRSF1 seems to play such a role in regulating SRA1 splicing, after SRSF1 binds to SRA1pre-mRNA, it recruits U1-U6 to bind to Pre-mRNA, increases the use of proximal 3′and 5′, and promotes the inclusion of SRA1 exon 3. Alternative splicing of pre-mRNA is a complex process, and many cofactors are involved. In the splicing process of SRA1, SRSF1 may play a major role, and it also needs other cofactors to complete the splicing of SRA1 together. So, beyond the involvement of SRSF1, other splicing factors may regulate the splicing of SRA1.

In addition, our data show that the binding ability of SRA1-L with PPAR-γ is stronger than that of *SRA1*-S (Fig. S4C, D). The PPAR-γ binding element (PPRE) exists in the *CD36* promoter region and enadbles PPAR-γ to transcriptionally regulate *CD36*^40^. The article reported that the core sequence of PREE in the *CD36* promoter is AAGTCA-G-AGGTCA. Yet, the core sequence of PREE is not fixed, unlike GGGGGA-A-AGGTTA in the *Cidea* promoter and AGGGCA-C-AGGAGA in *Fatp1*^41^. Through sequence comparison, we found that there are similar sequences in *CD44*, for example: AGGGCA-G-AGCTGG, AGGGCA-A-CATCAG, and GGGGGA-C-TGGAGT. We speculate that *SRA1-L* can regulate the transcription of *CD44* by binding to PPAR-γ. This will be the focus of our follow-up research.

In summary, our study identified an important oncogene, SRSF1, and an abnormal alternative splicing event, inclusion or skipping of SRA1 exon 3. Both isoforms of SRA1 play vital roles in cell invasion and migration and serve as prognostic biomarkers and therapeutic targets for liver cancer.

## Materials and methods

### Clinical specimens and cell cultures

Clinical research was performed according to written approval obtained from the first affiliated hospital of Jinan University (Guangzhou, China). HCCs and their matched adjacent noncancerous tissues were provided by the first affiliated hospital of Jinan University (Guangzhou,China). All clinical specimens were collected with the informed consent of the patients. Human embryonic kidney cell (293 T), human liver cell (LO2), and HCC cell lines (HepG2, Huh-7) were provided and identified by Guangzhou Institute of Biomedicine and Health. Human high metastatic HCC cells cells (HCCLM3) were purchased from Keygene Biotechnology Company Limited. HepG2, Huh-7, and HCCLM3 cells were cultured in RPMI-1640 (Gibco, Carlsbad, CA, USA), 293 T cells were maintained in DMEM (Gibco, Carlsbad, CA, USA). All media were supplemented with 10% FBS (Gibco, Carlsbad, CA, USA) and 1% penicillin/streptomycin (Beyotime, China). All cells were cultured at 37 °C in an atmosphere containing 5% CO_2_.

### Bioinformatics analysis

Bioinformatics web Rapmap^42^, RBpdb^43^, ESEfinder(c)^44,45^ were used to predict potential *SRSF1* binding sites on *SRA1* pre-mRNA. In addition, we predicted the coding potential of *SRA1-S* by CPAT^46^. The UALCAN database^24^ was employed to predict the correlation between the expression of different genes and liver cancer stages.

### Plasmid construct

The coding sequences (CDS) of SRSF1 (GenBank accession no. NM_006924), SRSF8 (GenBank accession no. NM_032102) and SRSF11 (GenBank accession nos. NM_001350605), and the full-length of *SRA1-S* and *SRA1-L* (GenBank accession no. NM_001035235) were PCR-amplified and subcloned into the pMD19-T Vector (TaKaRa, Japan), then subcloned into the *Pme* I site of the pMXs-Flag vector (Thermo Scientific, USA) named pMXs-*SRSF1*, pMXs–*SRSF8*, pMXs-*SRSF11*, pMXs-*SRA1-L*, pMXs-*SRA1-S* respectively. MS2–12× fragment was PCR-amplified by PrimeSTAR® HS from pSL-MS2–12× (Addgene, USA) using primers pcDNA3.1-MS2-HR-FP and pcDNA3.1-MS2-HR-RP. Next, homologous recombination kits were exploited to clone the fragment into *Eco*R V and *Xho* I site of pcDNA3.1 (+), named pcDNA3.1-MS2. The full-length of *SRA1-S*, *SRA1-L* was PCR-amplified from pMXs-*SRA1-L*, pMXs-*SRA1-S* using primers pcDNA3.1-SRA1-MS2-HR-FP and pcDNA3.1-SRA1-MS2-HR-RP, and cloned into *Xho* I site of the pcDNA3.1-MS2 vector using homologous recombination kits, named *SRA1-L*-MS2, *SRA1-S*-MS2. The Minigenes were constructed by amplifying genomic sequences spanning exons 2 to 4 of the SRA1 gene using primers SRA1-mini-HR-FP and SRA1-mini-HR-RP and, subsequently, using homologous recombination kits cloned the fragment into *Eco*R *V and Xho I* site of the pcDNA3.1 (+) vector, named SRA1-mini. MS2–12× fragment was PCR-amplified pSL-MS2–12× (Addgene, USA) using primers SRA1-mini-MS2-HR-FP/RP, subsequently, using homologous recombination kits cloned the fragment into *Xho I* site of SRA1-mini.

Short hairpin RNA (shRNA) sequences were designed to suppress the expression of SRSF1, SRSF8, SRSF11, SRA1-E3, SRA1-E2E4, and SRA1-In1. A scrambled shRNA was used as a negative control. After annealing, insert a double-stranded oligonucleotide between the *Hind* III and *Bgl* II restriction sites of the pSuper-Retro vector. The upstream and downstream primers were specifically designed to delete the RRMs and RS fragment of SRSF1. PCR amplification was carried out using pMXs-SRSF1 plasmid as a template. After adding *solution I* (TaKaRa, Japan) overnight connection, 1 μl *Dpn* I was added to 10 μl of the connection product, and after 1 h at 37 °C, 10 μl of the reaction product was 4 transformed into DH5α cells.

The primers employed in this paper were designed by our group with reference to the NCBI sequences and were synthesized by Guangzhou Qsingke Biological Company. The primers are shown in Supplementary Table. 1.

### Transfection and Retrovirus infection

All the plasmids were transfected into cells using Lipofectamine 6000 reagent (Beyotime, China) according to the manufacturer’s protocol. Retrovirus viral particles were produced in 293 T cells transfected with the pCL retroviral packaging plasmid in DMED media. Infectious lentivirus was harvested at 48 h post-transfection and filtered through 0.45 μm nitrocellulose filter. The viral supernatant was employed for infecting HepG2 or HCCLM3 cells after supplementation with 8 × 10^−6^ mg/ml Polybrene (Sigma-Aldrich, USA). The cells infected with viruses generated from transfection with pSUPER-Scramble (Scramble) or pMXs-Vector were used as control groups. For cells infected with shRNA retrovirus, cells with stable knockdown of the target genes could be obtained after 10 days of selection with 2 × 10^−3^ mg/ml Puromycin (Thermo Scientific, USA).

### Semi-quantitative RT-PCR and real-time qPCR (RT-qPCR) analysis

The Trizol (Beyotime, China) was applied to extract total RNA. Cytoplasmic and nuclear RNA was isolated and purified using the Nuclear and Cytoplasmic Protein Extraction Kit (Beyotime, China) according to the manufacturer’s instructions. Semi-quantitative RT-PCR was performed with Golden Star T6 Super PCR Mix (TsingKe, China), The PCR products were separated by 2% agarose gel electrophoresis. The ImageJ software was employed to quantify. Exon inclusion (%) = Gray value of long isomer / (gray value of long isomer + gray value of short isomer). Real-time qPCR was performed in the LightCyler 480 System (Roche, Switzerland) using SYBR® Premix Ex Taq™ (TaKaRa, Japan) and the gene-specific primers. *β-actin* was employed as an endogenous control. The relative expression of RNA was calculated using the 2^−ΔΔCt^ method.

### Cell proliferation assays

For cell proliferation assays, 3000 cells were seeded into 96-well plates. Cell proliferation was assessed using the CCK8 (Beyotime, China) according to the manufacturer’s protocol. Then OD_450_ values of each well were measured by Synergy TM HT (BioTeK, USA).

### Wound-healing assays and invasion assays

For wound-healing assays, linear scratch wounds (in triplicate) were created on the confluent cell monolayers using a 200 μl pipette tip. To remove cells from the cell cycle prior to wounding, cells were maintained in the serum-free medium. Scratch healing rate = (W_0 h_ -W_24 h_) ⁄ W_0 h_. The trans-well insert (Corning, USA) was ultized as per the manufacturer guideline. 3 × 10^5^ cells were added to the upper wells separated by an 8 μm pore size PET membrane with a thin layer of Matrigel basement membrane matrix for invasion. After being incubated 24 h, the non-migrated cells were removed from the top of the membrane with Cotton Cswab, and then the membranes were stained with DAPI (Beyotime, China) for 5 min after removing. Cells were visualized under a fluorescent microscope (ZEISS, AXIOVERT A1). Six random fields were captured at 200× magnification.

### Western blot analysis

The cells were lysed with RIPA buffer, and then the protein concentration was detected by BCA protein detection kit (Beyotime, China). 20 µg of whole-cell lysates was separated by SDS-PAGE and then transferred to a pre-activated Polyvinylidene Fluoride membrane (PVDF), the membrane was blocked for 1 h in TBST buffer (TBS containing 0.1% Tween 20) containing 5% non-fat dry milk followed by an overnight incubation with primary antibody diluted. AKT primary antibody (C67E7) and phosphorylated Ser473 AKT primary antibody (4060 T) were purchased from Cell Signaling Technology Company. ERK primary antibody (AF1051), phosphorylated Thr202 ERK primary antibody (AF1891) and β-actin primary antibody (AF0003) were purchased from Beyotime Company. SRSF1 primary antibody (NHA3445) was purchased from Novogene Company. After extensive washing, the blot was incubated with a secondary antibody overnight. Finally, the membranes were washed thrice with TBST and visualized by BeyoECL Moon kit (Beyotime, China). Imaging was then performed using a biochemi-luminometer (General Electric, AI600) and Image J software was ultized to quantify the bands’ grayscale.

### RNA pull-down assays

The RNA pull-down assays were carried out by synthesized biotinylated SRA1 RNA (Tsingke, China) as a probe. The Pierce™ Magnetic RNA-Protein Pull-Down Kit (Thermo Scientific, USA) was used to perform the experiment. For each sample, 50 μl streptavidin magnetic beads were engrafted to capture the biotin-labeled RNA. The products of RNA and nuclear protein complexes were washed and analyzed by western blot.

### RNA immunoprecipitation assays

pcDNA3.1-MS2-SRA1-mini, pcDNA3.1-MS2-*SRA1-L*, pcDNA3.1-MS2-*SRA1-S* and pcDNA3.1-MS2 were co-transfected with pMS2-GFP (Addgene, USA) into HepG2 cells. Cell lysates were utilized to perform RIP experiments using a GFP antibody (Beyotime, China) coupled with protein G magnetic beads (50 μl per reaction) 48 h later. Subsequently, 500 ul of RIPA was added to the magnetic bead complex. Finally, the protein was analyzed by western blot.

### Cross-link immunoprecipitation (CLIP)

CLIP was performed to certify the binding between SRSF1 and SRA1. Briefly, RNA and protein complexes were cross-linked with 1% formaldehyde in ultraviolet cross-linking was performed at 400 mJ cm^−2^ for 10 min. Immunoprecipitation was done with 10 μg anti-SRSF1 antibody (Novogene, China) or either nonspecific IgG coupled with 50 μl protein G magnetic beads. Then, extracting RNA from complexes and synthesizing cDNA with random primers. Finally, RNA enrichment was measured by RT–PCR with primers specific for SRA1 mRNA.

### Luciferase reporter assays

pGL3-CDH1, pGL3-CD44, pGL3-TP53, pGL3-CAV1, and phRL-SV40 were co-transfected into overexpressed or knocked down SRA1 isoform HepG2 cells by using Lipofectamine 6000 reagent (Beyotime, China) according to the manufacturer’s protocol. 48 h after transfection, the cells were lysed, and luciferase activity was measured using a Dual-Luciferase Reporter Gene Assay Kit (Beyotime, China).

### In vivo metastasis assays

3-week-old male nude mice (BALB/c-nu/nu) (*N* = 48) were purchased from Beijing HFK Biotechnology Co, Ltd. The animals were maintained under Specific Pathogen Free (SPF) conditions and were provided food and water. The animals were randomly divided into 6 groups with 8 animals in each group. Six different groups (sh-scramble, sh-*SRSF1*, sh-*SRA1-L*, sh-*SRSF1* + pMXs-flag, sh-SRSF1 + pMXs-*SRA1-L*, sh-*SRSF1* + pMXs-*SRA1-S*) of HCCLM3 cells (1 × 10^6^ cells in 100 μl serum-free media) were injected into the tail vein of 4-week-old BALB/C female nude mice (weighing 18–22 g, *N* = 48). After 30 days of normal feeding, lungs were harvested at necropsy and fixed in paraformaldehyde. All animal procedures were performed in accordance with the Jinan University Experimental Animal Care Commission. All animal experiments comply with ARRIVE guidelines, and are carried out in accordance with the U.K. Animals (Scientific Procedures) Act, 1986, EU Directive 2010/63/EU for animal experiments, and the National Institutes of Health guide for the care and use of Laboratory animals (NIH Publications No. 8023, revised 1978). No blinding was used in animal experiments.

### HE (Haematoxylin-Eosin)

The lungs were fixed in 4% paraformaldehyde and transferred to 70% ethanol, then embedded in paraffin. Before immunostaining, 4-µm-thick lung tissue sections were dewaxed in xylene, rehydrated through decreasing concentrations of ethanol, and washed in PBS. Then stained with hematoxylin and eosin (H&E). After staining, sections were dehydrated through increasing concentrations of ethanol and xylene.

### Immunohistochemistry (IHC)

For immunohistochemical analysis of E-Cadherin, N-Cadherin tissue sections were prepared, as described previously^47^. The primary antibody used was E-Cadherin Mouse Monoclonal Antibody (1:100) (Beyotime, AF0138) and N-Cadherin Rabbit Polyclonal Antibody (1:200) (Beyotime, AF0138).

### Statistics analysis

Using SPSS Statistical Package version 24 (SPSS Inc, Chicago, Illinois) to analyze the correlation of data. Data from the experiments with one experimental group were presented as means ± Standard Deviation (SD) (all experiments were performed in triplicate or more). One-way (or two-way) analysis of variance (ANOVA) and Tukey’s post-hoc analysis was exploited to evaluate statistical significance. *P < 0.05, **P < 0.01 or ***P < 0.001 was considered to indicate statistical significance, “#” represents *P* < 0.05, “##” represents *P* < 0.01, and “###” represents *P* < 0.001, ns means no significance. When representative figures are shown, these are representative of three independent repeats.

## Supplementary information

Table S1

table legends

Supplementary figure legends

STR-hepg2

figS1

figS2

figS3

figS4

figS5
