# Supplementary material for: SRSF1 promotes the inclusion of exon 3 of SRA1 and the invasion of hepatocellular carcinoma cells by interacting with exon 3 of SRA1pre-mRNA
Source: Cell Death Discov. 2021 May 19;7:117. doi: 10.1038/s41420-021-00498-w (PMC8134443; doi:10.1038/s41420-021-00498-w)
Supplement: Supplementary file 1 — Table S1 [file 41420_2021_498_MOESM1_ESM.docx]

**Supplementary Table S1 List of primers**

| SRSF1-ORF-FP | 5'ATGTCGGGAGGTGGTGTGATTC3' |
| --- | --- |
| SRSF1-ORF-RP | 5'TGTACGAGAGCGAGATCTGCTATGA3' |
| SRSF8-ORF-FP | 5'GCTCTCGGAGCCATGAGCTG3' |
| SRSF8-ORF-RP | 5'TTAAGAGGACATCTGTCCTTCCTCT3' |
| SRSF11-ORF-FP | 5'ATGAGCAACACTACCGTCGT3' |
| SRSF11-ORF-RP | 5'TCAGTCACTCATATCCATGTCTTCT3' |
| SRSF1-HR-FP | 5'GTTAATTAAGGATCCGTTTGCCACCATGTCGGGAGGTGGTGTGA3' |
| SRSF1-HR-RP | 5'CGTCCTTGTAGTCTTGTTTTGTACGAGAGCGAGATCTGCTA3' |
| SRSF8-HR-FP | 5'GTTAATTAAGGATCCGTTTGCCACCGCTCTCGGAGCCATGAG3' |
| SRSF8-HR-RP | 5'CGTCCTTGTAGTCTTGTTTAGAGGACATCTGTCCTTCCTCT3' |
| SRSF11-HR-FP | 5'GTTAATTAAGGATCCGTTTGCCACCATGAGCAACACTACCGTCGT3' |
| SRSF11-HR-RP | 5'CGTCCTTGTAGTCTTGTTTGTCACTCATATCCATGTCTTCT3' |
| SRA1-FP | 5‘GGAGATGGCGGAGCTGTAC3’ |
| SRA1-RP | 5‘ATGAGGCAGCAGTTTGTTTGGCAAG3’ |
| SRA1-HR-FP | 5'GTTAATTAAGGATCCGTTTGCCACCGGAGATGGCGGAGCTGTAC3' |
| SRA1-HR-RP | 5'CGTCCTTGTAGTCTTGTTTAGGCAGCAGTTTGTTTGGCAAG3' |
| sh-SRSF1-1^st^ | 5'gatcccc GAAGCAGGTGATGTATGTTAT ttcaagaga3' |
| sh-SRSF1-2^nd^ | 5’ATAACATACATCACCTGCTTC ttttta3' |
| sh-SRSF1-3^rd^ | 5’ ATAACATACATCACCTGCTTCggg3' |
| sh-SRSF1-4^th^ | 5'agcttaaaaa GAAGCAGGTGATGTATGTTATtctcttgaa3' |
| sh-SRSF8-1^st^ | 5'gatcccc CCACTAGCTCTCGCTCTGCAT ttcaagaga3' |
| sh-SRSF8-2^nd^ | 5’ATGCAGAGCGAGAGCTAGTGG ttttta3' |
| sh-SRSF8-3^rd^ | 5’ATGCAGAGCGAGAGCTAGTGGggg3' |
| sh-SRSF8-4^th^ | 5'agcttaaaaa CCACTAGCTCTCGCTCTGCATtctcttgaa3' |
| sh-SRSF11-1^st^ | 5'gatcccc GGGAACTGGTGATTCACTAAG ttcaagaga3' |
| sh-SRSF11-2^nd^ | 5’CTTAGTGAATCACCAGTTCCC ttttta3' |
| sh-SRSF11-3^rd^ | 5’CTTAGTGAATCACCAGTTCCCggg3' |
| sh-SRSF11-4^th^ | 5'agcttaaaaa GGGAACTGGTGATTCACTAAGtctcttgaa3' |
| sh-SRA1-E3-1^st^ | 5'gatcccc ACAAGTTTCCCAGTCGAGTCT ttcaagaga3' |
| sh-SRA1-E3-2^nd^ | 5’AGACTCGACTGGGAAACTTGTttttta3' |
| sh-SRA1-E3-3^rd^ | 5’AGACTCGACTGGGAAACTTGTggg3' |
| sh-SRA1-E3-4^th^ | 5'agcttaaaaa ACAAGTTTCCCAGTCGAGTCT tctcttgaa3' |
| sh-SRA1-E2E4-1^st^ | 5'gatcccc CCACACAAGGAAGCAGGTATGttcaagaga3' |
| sh-SRA1-E2E4-2^nd^ | 5’CATACCTGCTTCCTTGTGTGGttttta3' |
| sh-SRA1-E2E4-3^rd^ | 5’CATACCTGCTTCCTTGTGTGGggg3' |
| sh-SRA1-E2E4-4^th^ | 5'agcttaaaaa CCACACAAGGAAGCAGGTATG tctcttgaa3' |
| sh-SRA1-In1-1st | 5'gatcccc CAGTATAAGCTAACAGTGGAG ttcaagaga3' |
| sh-SRA1-In1-2nd | 5’CTCCACTGTTAGCTTATACTGttttta3' |
| sh-SRA1-In1-3rd | 5’CTCCACTGTTAGCTTATACTGggg3' |
| sh-SRA1-In1-4th | 5'agcttaaaaa CAGTATAAGCTAACAGTGGAG tctcttgaa3' |
| SRSF1-RRM1-FP | 5'GGCCGTGGAACAGGCCGAG3' |
| SRSF1-RRM1-RP | 5'ATCGTTGTTCCCTGCGGGGC3' |
| SRSF1-RRM2-FP | 5'GGGCCCAGAAGTCCAAGTTAT3' |
| SRSF1-RRM2-RP | 5' TTCAGACCGCCTGGATGGG3' |
| SRSF1-RS-FP | 5'ACAAAACAAGACTSCAAGGAC3' |
| SRSF1-RS-RP | 5'GGGCCCATCAACTTTAACC3' |
| pcDNA3.1-SRA1-MS2-HR-FP | 5'TGGTGGAATTCTGCAGATATCGGAGATGGCGGAGCTGTAC3' |
| pcDNA3.1-SRA1-MS2-HR-RP | 5'GATCCATATATAGGGCCCGGGATATCATGAGGCAGCAGTTTG3' |
| pcDNA3.1-MS2-HR-FP | 5'TGGTGGAATTCTGCAGATATCCCGGGCCCTATATATGGATC3' |
| pcDNA3.1-MS2-HR-RP | 5’AACGGGCCCTCTAGACTCGAGTCGATCGCGCGCAGATCTAA3’ |
| SRA1-mini-FP | 5‘GGAGATGGCGGAGCTGTAC3’ |
| SRA1-mini-RP | 5‘CTTGCACCAGTAGAGCCATTCTCTT3’ |
| SRA1-mini-HR-FP | 5’TGGTGGAATTCTGCAGATATCGGAGATGGCGGAGCTGTACGTGA3’ |
| SRA1-mini-HR-RP | 5’AACGGGCCCTCTAGACTCGAGCTTGCACCAGTAGAGCCATTCTCTT3’ |
| SRA1-mini-MS2-HR-FP | 5’TGGCTCTACTGGTGCAAGCTCGAGCCGGGCCCTATATATGGATC3’ |
| SRA1-mini-MS2-HR-RP | 5’AACGGGCCCTCTAGACTCGAGTCGATCGCGCGCAGATCTAA3’ |
