# Supplementary material for: SRSF1 promotes the inclusion of exon 3 of SRA1 and the invasion of hepatocellular carcinoma cells by interacting with exon 3 of SRA1pre-mRNA
Source: Cell Death Discov. 2021 May 19;7:117. doi: 10.1038/s41420-021-00498-w (PMC8134443; doi:10.1038/s41420-021-00498-w)
Supplement: Supplementary file 2 — table legends [file 41420_2021_498_MOESM2_ESM.docx]

table legends

Supplementary table S1: List of primers. NCBI (National Center for Biotechnology Information) was used to find the genome and mRNA sequence of the gene. According to the nucleic acid sequence, primers were designed with primer5.0.
