# Supplementary material for: SRSF1 promotes the inclusion of exon 3 of SRA1 and the invasion of hepatocellular carcinoma cells by interacting with exon 3 of SRA1pre-mRNA
Source: Cell Death Discov. 2021 May 19;7:117. doi: 10.1038/s41420-021-00498-w (PMC8134443; doi:10.1038/s41420-021-00498-w)
Supplement: Supplementary file 3 — Supplementary figure legends [file 41420_2021_498_MOESM3_ESM.docx]

**Supplementary Figure legends**

**Supplementary Fig. S1.** SRSF1 is the Major Regulator for *SRA1* exon 3 Splicing. (A) Different expression levels of splicing factor in human HCC tissues from TCGA samples. (B) Western blot was used to detect the differential expression of SRSF1 in LO2, HepG2, Huh-7 and HCCLM3 cells. Experiments with two experimental groups were statistically analyzed using Two-tailed Student’s t-tests. The “*, **, ***” indicates “P<0.05, 0.01, 0.001” versus the control group, respectively. "#" represents P<0.05, "##" represents P<0.01, and "###" represents P<0.001.

**Supplementary Fig. S2.** SRSF1, SRSF8, SRSF11 overexpression and knockdown efficiency detection. (A) Detection overexpression efficiency SRSF1, SRSF8, SRSF11 in HCCLM3 by RT-qPCR. (B) Detection knockdown efficiency SRSF1, SRSF8, SRSF11 in HCCLM3 by RT-qPCR. Experiments with two experimental groups were statistically analyzed using Two-tailed Student’s t-tests. The “*, **, ***” indicates “P<0.05, 0.01, 0.001” versus the control group, respectively.

**Supplementary Fig. S3.** SRSF1 overexpression and knockdown efficiency detection. (A) Detection overexpression efficiency SRSF1 by Western-blot (B) Detection knockdown efficiency SRSF1, SRSF8, SRSF11 in HCCLM3 by Western-blot. (C-D) Quantitative analysis. Experiments with two experimental groups were statistically analyzed using Two-tailed Student’s t-tests. The “*, **, ***” indicates “P<0.05, 0.01, 0.001” versus the control group, respectively.

**Supplementary Fig. S4.** The effect of the SRSF1 domain on the downstream target gene. (A) Detection of the effects of SRSF1 domains on alternative splicing of SRA1. (B) CCK8 assays were carried out to examine the effect on cell proliferation of SRSF1 in HepG2 and HCCLM3. (C, D) The effect of SRA1 bind to PPARγ was investigated by the MS2-GFP-IP system.

**Supplementary Fig. S5.** SRA1-L/S overexpression and knockdown efficiency detection. (A) Detection overexpression efficiency SRA1-L/S by RT-PCR. (B) Detection knockdown efficiency SRA1-L/S by RT-PCR. Experiments with two experimental groups were statistically analyzed using Two-tailed Student’s t-tests. The “*, **, ***” indicates “P<0.05, 0.01, 0.001” versus the control group, respectively.
