# Supplementary figures and images for: SRSF1 promotes the inclusion of exon 3 of SRA1 and the invasion of hepatocellular carcinoma cells by interacting with exon 3 of SRA1pre-mRNA
Source: Cell Death Discov. 2021 May 19;7:117. doi: 10.1038/s41420-021-00498-w (PMC8134443; doi:10.1038/s41420-021-00498-w)

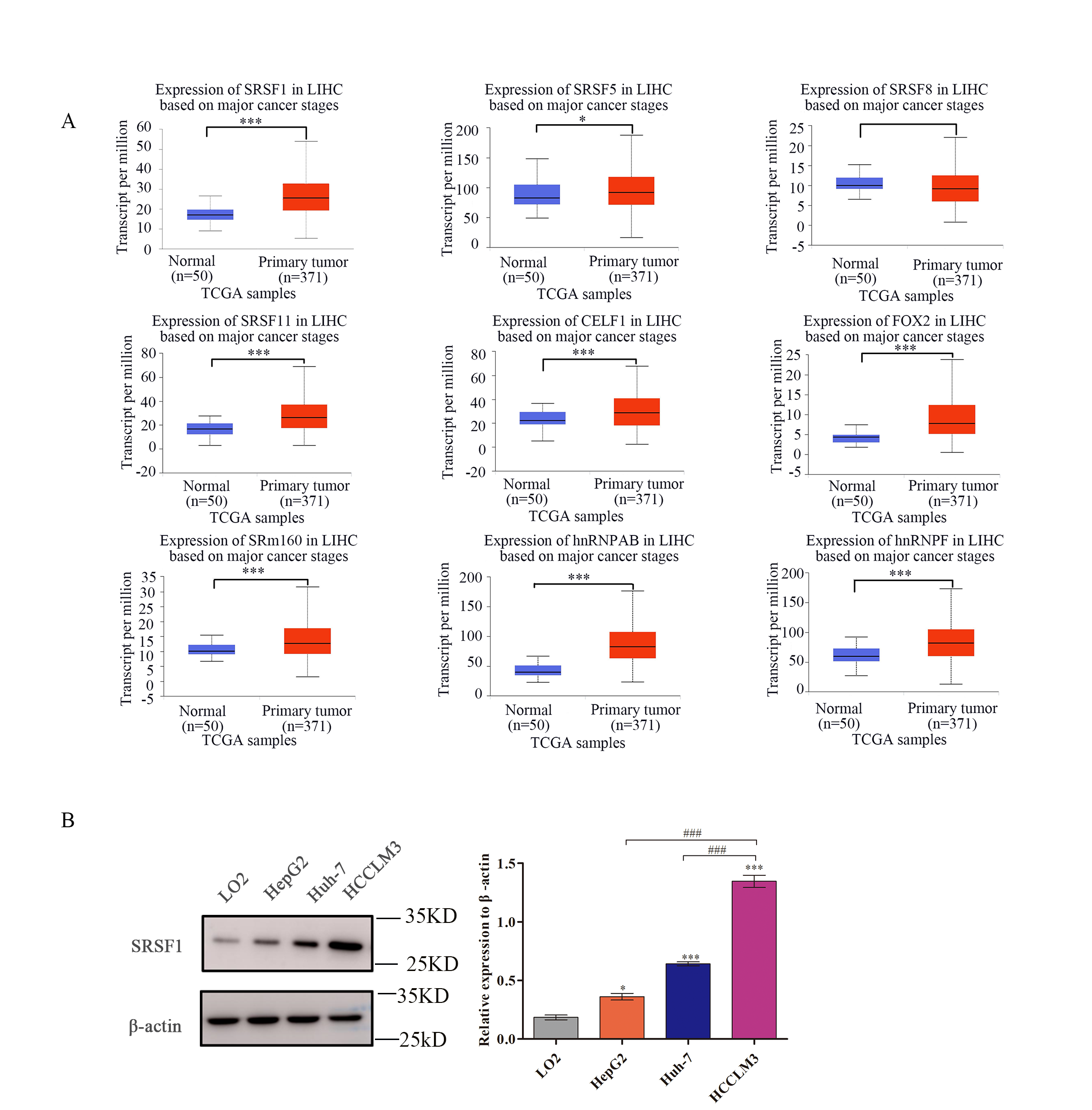

Supplement: Supplementary file 5 — figS1 [file 41420_2021_498_MOESM5_ESM.tif]

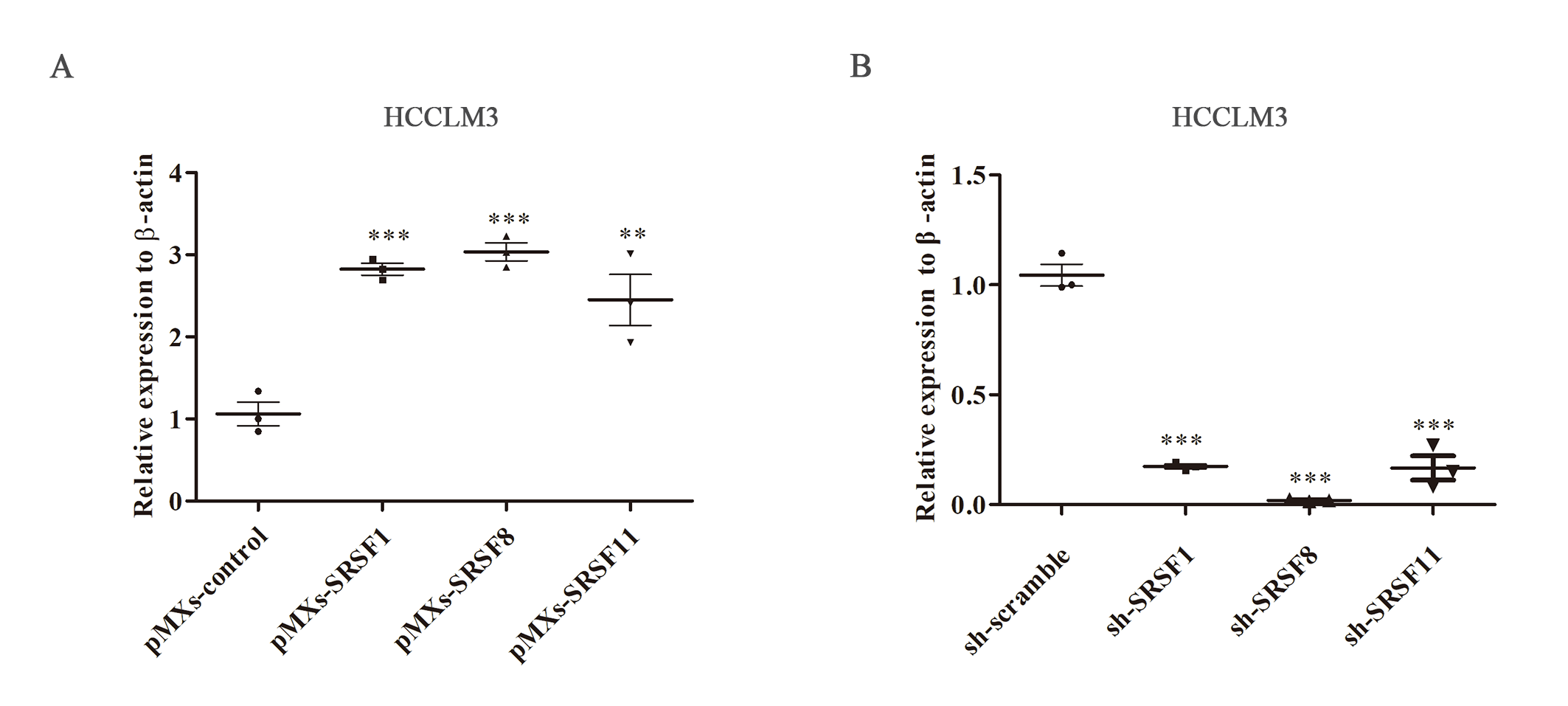

Supplement: Supplementary file 6 — figS2 [file 41420_2021_498_MOESM6_ESM.tif]

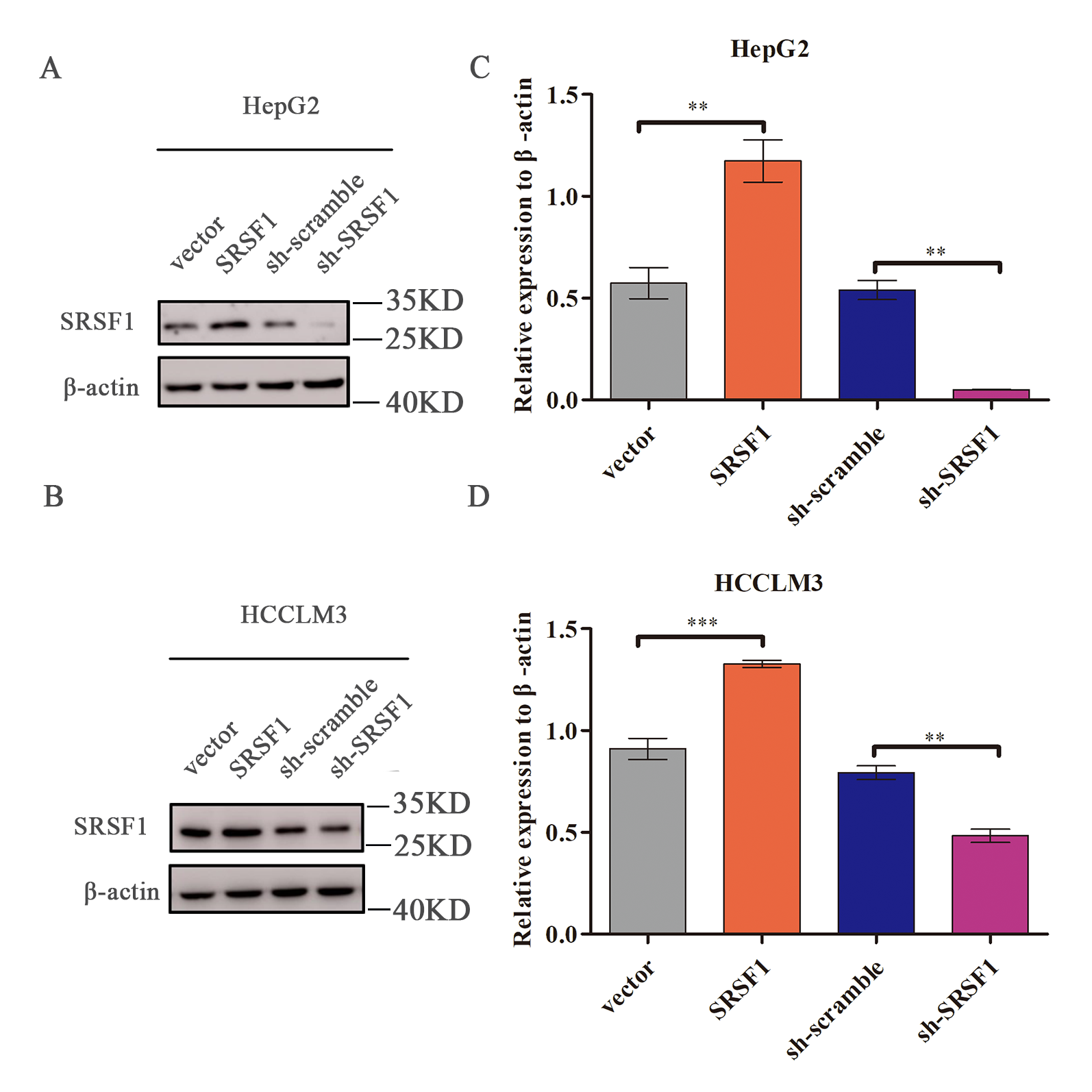

Supplement: Supplementary file 7 — figS3 [file 41420_2021_498_MOESM7_ESM.tif]

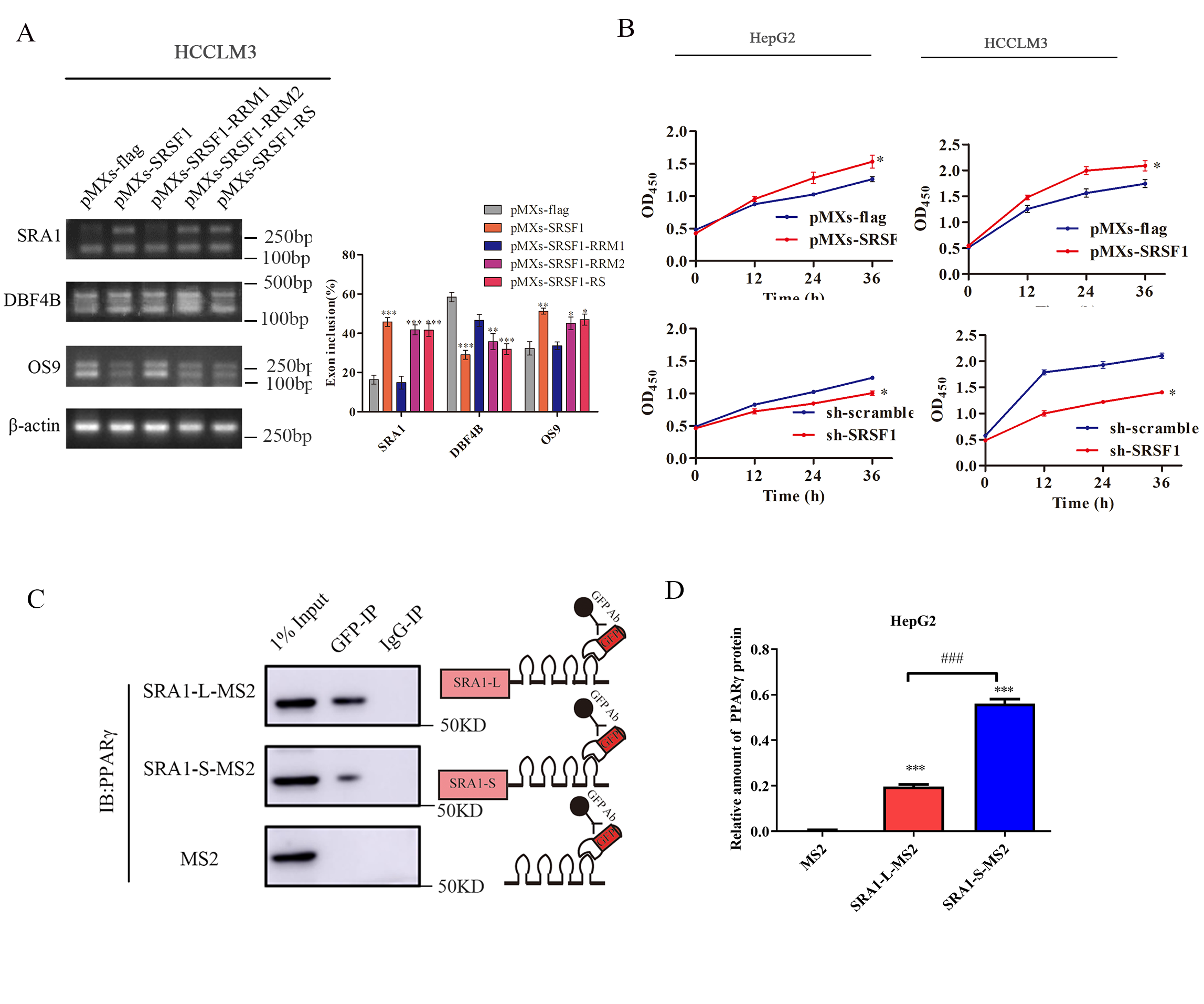

Supplement: Supplementary file 8 — figS4 [file 41420_2021_498_MOESM8_ESM.tif]

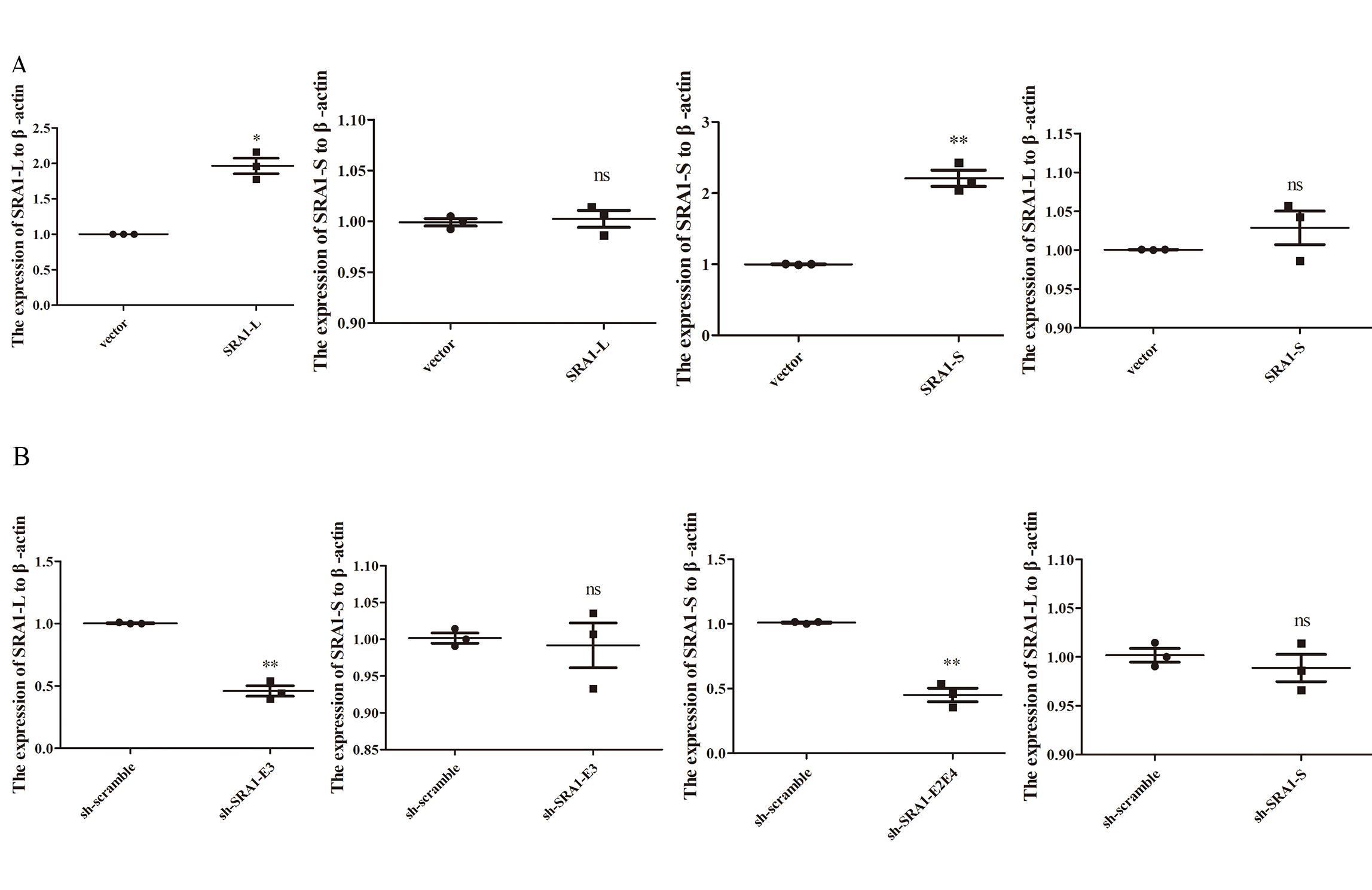

Supplement: Supplementary file 9 — figS5 [file 41420_2021_498_MOESM9_ESM.tif]
